# From Compensation to Collapse: UVB-Driven Disruption of Host–Microbiota Homeostasis Exacerbates Amphibian Ecological Risk

**DOI:** 10.3390/ani15223236

**Published:** 2025-11-07

**Authors:** Zi’ao Yuan, Jirui Fei, Siqi Li, Yueluan Wu, Peng Liu

**Affiliations:** College of Life Science and Technology, Harbin Normal University, Harbin 150080, China; yiwuanemail@126.com (Z.Y.); 18846420279@163.com (J.F.);

**Keywords:** amphibian, ultraviolet radiation, oxidative stress, symbiotic microbiota, gut-skin axis

## Abstract

The synergistic effects of stratospheric ozone depletion and climate change are intensifying surface ultraviolet-B (UVB) radiation, posing a severe threat to amphibians—one of the most endangered vertebrate groups globally. *Xenopus laevis*, with its cutaneous respiration and limited photoprotective mechanisms, exhibits high sensitivity to UVB, making it a suitable model for ecotoxicological studies. While UVB is known to cause DNA damage, immune suppression, and microbial dysbiosis, its mechanisms in multi-organ interactions, dose–response thresholds, and host–microbiome regulatory networks remain poorly understood. This study employed a gradient UVB exposure regime integrated with histopathology, oxidative stress assays, and 16S rRNA sequencing to systematically evaluate the effects of UVB on (1) cascade damage across skin, liver, and intestinal barriers; (2) immune cell distribution; (3) redox dynamics; and (4) microbial community structure and function. Our findings demonstrate that low-dose UVB activated compensatory antioxidant defenses without structural disruption, whereas exposure beyond a critical threshold induced nonlinear redox collapse, microbial dysbiosis, and multi-organ barrier failure, collectively exacerbating ecological adaptation risks. These results reveal a cross-scale mechanism by which UVB impairs amphibian health via disruption of host–microbe homeostasis, providing a conceptual and empirical framework for assessing species vulnerability under ongoing climate change.

## Simple Summary

Amphibians face severe threats from increasing ultraviolet-B (UVB) radiation due to ozone depletion and climate change. Using *Xenopus laevis* as a model, this study investigated the effects of varying UVB doses on multi-organ health and symbiotic microbiota. We found that low-dose UVB activated protective responses, including elevated antioxidant enzyme activities and proliferation of anti-inflammatory bacteria. However, beyond a specific threshold, UVB exposure induced a collapse of the redox defense system, damaged skin and gut barriers, and led to microbial dysbiosis. Collectively, these changes exacerbated physiological stress and compromised the host’s ecological adaptability. Our study reveals a cross-organ mechanism through which UVB disrupts host–microbe homeostasis, thereby threatening amphibian survival. These findings provide a scientific basis for predicting population decline risks under ongoing climate change and offer insights for developing targeted conservation strategies, such as habitat shade management and microbiota intervention.

## 1. Introduction

During the past several decades, the synergistic effects of stratospheric ozone depletion and climate change have exacerbated surface ultraviolet-B (UVB, 280–315 nm) radiation, with its ecotoxicological consequences emerging as a critical challenge to global biodiversity [1]. While the Montreal Protocol has effectively curbed the expansion of the ozone hole, UVB flux in high-latitude/altitude habitats continues to increase [2], imposing dual survival pressures on amphibians—the most endangered vertebrate group globally (41% of species) [3]. Their unique physiological traits, including biphasic aquatic–terrestrial life cycles reliant on cutaneous respiration, poikilothermic sensitivity to microclimate variability, and a lack of photoprotective barriers (e.g., melanin or keratinized strata), render amphibians particularly vulnerable to photodamage during periods of peak UVB exposure (spring–summer) [4]. Both field and laboratory studies have demonstrated that UVB acts as a sublethal stressor capable of inducing multi-level damage in fish and other aquatic vertebrates: prior to the manifestation of phenotypes such as embryonic developmental abnormalities and reduced locomotor performance, it can trigger molecular pathological changes, including DNA damage and immunosuppression [1,5,6], ultimately leading to population decline and disruption of community structure. Due to their physiological characteristics, amphibians exhibit heightened sensitivity to UVB and are thus more severely affected. The amphibian innate immune system employs a multi-tiered defense cascade: (1) physical barriers (integumentary and intestinal structural integrity), (2) immune cellular defenses (Th1/Th2 balance), and (3) microbial barriers (symbiotic microbiota colonization resistance). Recent studies have demonstrated that UVB systematically disrupts these mechanisms: epidermal keratinocyte apoptosis compromises physical barriers [7]; aberrant mast cell degranulation triggers Th2 polarization and chronic inflammation [8]; and cutaneous/intestinal dysbiosis impairs anti-pathogen resistance [7]. Notably, the pathological interplay of the “gut–skin axis” warrants increased emphasis. As primary microbial reservoirs, these organs maintain a dynamic equilibrium via neuroendocrine–immune networks [9]. UVB-induced intestinal hyperpermeability exacerbates cutaneous oxidative damage, while cutaneous dysbiosis reciprocally disrupts gut immune homeostasis via circulatory pathways, thereby establishing a pathogenic positive feedback loop [10].

Beyond its immunoregulatory roles, the skin serves as a crucial physical and biochemical barrier in vertebrates, employing selective absorption of ultraviolet radiation (UVR) for photoprotection. However, intense UVB disrupts the functionality of the gut–skin axis, significantly increasing the risk of pathogen invasion [5]. Amphibian larvae exhibit heightened UVB sensitivity due to dermal developmental heterogeneity and immune ontogeny asynchrony [8]. Systemic understanding of UVB’s acute effects in vertebrates includes epidermal hyperplasia and melanogenesis as photoprotective responses, along with structural compromises manifesting as reduced tensile strength, aberrant keratinization, and dermal collagen fragmentation [11]. While UVB-triggered mucous cell hypertrophy transiently enhances barrier function, excessive responses impair critical physiological processes such as respiration, excretion, and osmoregulation [8,12]. Such imbalances in a dynamic barrier significantly increase the susceptibility to fungal infection [13], suggesting that UVB affects host defenses through dual mechanisms: physical barrier disruption and immunoregulatory dysfunction.

At the immunoregulatory level, mast cells—key effectors in mucocutaneous immunity—are a critical link between degranulation and UVB-induced immunosuppression. Studies have indicated that UVB alters mast cell phenotypes/spatial distributions, modulating the temporal release of histamine and cytokines [14]. This immune network dysregulation likely disrupts the Th1/Th2 equilibrium, thereby perpetuating inflammation and tissue remodeling anomalies [8]. Importantly, a dynamic synergy may exist between mucopolysaccharide-antimicrobial peptide secretion and mast cell-mediated immunity, with system homeostasis being pivotal for amphibian pathogen resistance [9]. UVB’s biological effects exhibit multi-organ interconnectivity. Emerging evidence suggests that impairment of the epidermal barrier may compromise intestinal epithelial adhesion [15], while hepatocyte responses to UV stress could serve as biomarkers for systemic immune status [16]. Elucidating such cross-organ interactions will advance our understanding of the role of environmental stressors in global amphibian declines.

UVB genotoxicity primarily stems from oxidative stress via direct photolysis or indirectly via the generation of reactive oxygen species (ROS) inducing DNA damage, lipid peroxidation, and protein denaturation [17]. Amphibians’ complex life histories increase their vulnerability to UV, with UVB reducing survival rates by 1.9-fold [4]. Malondialdehyde (MDA), a lipid peroxidation end-product, reflects the severity of membrane damage, while superoxide dismutase (SOD) and catalase (CAT)—key antioxidant enzymes—mitigate oxidative injury by scavenging radicals [18]; both enzymes thus serve as environmental stress biomarkers [19]. Notably, UVB-induced oxidative damage exhibits a threshold effect: acute high-dose exposure triggers a surge in ROS and collapse of antioxidant capacity, whereas low-dose radiation may activate adaptive responses [20]. However, the cumulative effects of chronic low-dose exposure and its interplay with immunosuppression remain unexplored.

Emerging evidence implicates UVB in amplifying ecological risks through host–microbe–environment networks [10]: the loss of epidermal integrity alters the cutaneous microenvironment, reducing antifungal peptide-secreting microbiota (e.g., *Micrococcus luteus*) [21,22], while the decline in gut microbial β-diversity exacerbates systemic inflammation via the gut–skin axis [23]. Pathogen enrichment (e.g., *Aeromonas hydrophila*) under UVB stress is correlated with diminished immune surveillance [24]. Specific cases in high-altitude habitats show that enhanced UVB radiation increases amphibian susceptibility to *Batrachochytrium dendrobatidis* [5], indicating that microbiome disturbance may play a key role in the decline of amphibian populations.

Although considerable progress has been made in understanding the effects of UVB on individual tissues, critical knowledge gaps remain regarding multi-organ interactions, daily cumulative irradiance and time-dependent kinetics, and host–microbiome network regulation. Using *Xenopus laevis* as a model organism and a gradient UVB exposure system, this study systematically investigated the changes induced by increasing UVB intensity in the following aspects: (1) the cascade damage pattern of the skin–liver–gut barrier; (2) the distribution dynamics of immune cells (mucous cells and mast cells); (3) the response characteristics of oxidative stress levels (SOD/CAT-MDA system); and (4) the structural and functional shifts in microbial diversity. We proposed and tested two core hypotheses:

**H_1_:**  *Low-intensity UVB activates compensatory antioxidant responses without causing structural damage, whereas high-intensity UVB induces nonlinear collapse of the redox system.*

**H_2_:**  *High-intensity UVB radiation leads to markedly different community structures in the skin and gut microbiota, and specific symbiotic dysbiosis is closely associated with host pathology. By integrating histopathology, redox analysis, and 16S rRNA sequencing, this study elucidates the cross-scale mechanisms through which UVB threatens amphibian survival via host–microbe interactions, thereby establishing a new paradigm for assessing climate change-driven ecological risks.*

## 2. Materials and Methods

### 2.1. Animal Husbandry and Management

Subadult *Xenopus laevis* were obtained from a standard breeding facility in Harbin, Heilongjiang Province, China (47.646° N, 130.343° E; elevation 98 m). The animals were acclimatized under laboratory conditions (photoperiod: 12L:12D; temperature 25 ± 1 °C) for ≥2 weeks in a recirculating aquaculture system with biological filtration. Dechlorinated tap water (residual chlorine < 0.01 mg/L) was partially replaced every 48 h to maintain dissolved oxygen levels. Sterile-cultured *Limnodrilus hoffmeisteri* was provided with a sterile fixed daily ration, and excreta was promptly removed to maintain hygienic conditions. All procedures strictly adhered to the guidelines approved by the Experimental Animal Ethics Committee of Harbin Normal University (IACUC: HNUARIA 2022002) and complied with the 3R principles (Replacement, Reduction, and Refinement).

### 2.2. Experimental Design and Irradiation Protocol

In natural environments, the duration of sunlight (photoperiod) varies predictably with latitude and season, leading to differences in the daily cumulative UVB irradiance experienced by animals. To simulate this natural gradient, this study employed a UVB irradiance of 0.5 W/m^2^ (REP-SHOP UVB 15W/12 lamp, Hunan province, China) and established three UVB exposure groups (U6H, U12H, U18H) with daily exposure durations of 6, 12, and 18 h, corresponding to daily cumulative irradiances of 10.8, 21.6, and 32.4 kJ/m^2^, respectively. This design creates an experimental gradient from short to typical and finally to long photoperiods. To define the UVB exposure safety threshold, a preliminary experiment was conducted. An extreme long-duration exposure of 18 h/day, simulating the prolonged sunlight conditions of high-latitude summers or seasons with persistent strong illumination, was administered continuously for 30 days. This regimen induced severe skin ulcers (incidence >90%) and significant mortality in *Xenopus*. Consequently, this duration was defined as the maximum exposure period for the formal experiment. In the formal experiment, based on animal welfare considerations (adhering to the 3R principles) and the goal of improving survival rates, the illumination protocol for the U18H group was optimized to intermittent exposure (6 h of irradiation followed by a 2 h interval). A visible-light control group (15 W incandescent lamp, 400–700 nm spectrum) was concurrently maintained. The UVB output was calibrated using a PMA2100 radiometer (Solar Light^TM^, Glenside, PA, USA). To maintain precise irradiance levels and exclude potential confounding effects from non-UVB thermal radiation, the light source was vertically positioned 45 cm above the dorsal surface of the animals. The water volume in each rearing tank was strictly standardized to ensure consistent heat capacity across all groups. The water surface temperature was monitored daily after irradiation using an infrared thermometer (22 °C). No significant differences were detected among any experimental groups, confirming that the observed biological effects were attributable to specific UVB exposure rather than differential thermal stress.

*Xenopus* (*n* = 40/group; body weight, 4.17 ± 0.03 g; snout–vent length, 32.88 ± 0.09 mm; and sex ratio, 1:1) were randomly housed in polyethylene tanks (50 × 37.5 × 29 cm). At days 7, 15, 21, and 30 post-irradiation, a subset of animals (n = 10 per group per time point, representing biological replicates) were euthanized via double-pithing. Skin (SK), liver (LI), and large intestine (LA) samples were aseptically collected. The tissues were rinsed with ice-cold Ringer’s solution and immediately stored at −80 °C for subsequent analyses.

### 2.3. Histomorphological Analysis

Fresh tissues were fixed in Bouin’s solution for 48 h and processed using a standard protocol described by Liu and Liu [25] that involved ethanol dehydration, xylene clearing, and paraffin embedding. Serial 5-μm sections were stained with hematoxylin-eosin (H&E) and digitized for pathological evaluation of the epidermal thickness, cellular architecture, and inflammatory infiltration. Special stains included aldehyde fuchsin-orange G (mast cells) and Alcian blue-periodic acid Schiff (AB-PAS; mucous cells). Ten non-overlapping fields per sample were randomly imaged.

### 2.4. Oxidative Stress Biomarker Assays

Frozen skin and liver tissues were homogenized (10% *w*/*v* in ice-cold buffer) and centrifuged (4 °C, 3000× *g*, 10 min). The supernatants were analyzed for total protein (TP), malondialdehyde (MDA), superoxide dismutase (SOD), and catalase (CAT) using commercial kits (Nanjing Jiancheng Bioengineering Institute, Nanjing, China) and a SpectraMax M5 microplate reader (San Jose, CA, USA). Triplicate technical replicates were performed.

### 2.5. Microbiome Profiling

At the experimental endpoint (Day 30), skin swabs (a sterile swab was repeatedly scraped across the dorsal skin 10 times) and intestinal contents (luminal extrusion) [26,27] were collected. Due to the small body size of *Xenopus*, the microbial biomass obtainable from a single individual was insufficient for reliable downstream analysis. To address this, a pooling strategy was employed to meet the required biomass input while maintaining biological replication (n = 3 biological replicates per group, with each replicate derived from 3 individuals). All samples were immediately stored at −80 °C until further analysis. The control skin and fecal samples were referred to as CS and CF, respectively, and the UVB-treated skin and fecal samples as US and UF, respectively.

Total genomic DNA was extracted from the skin and fecal samples using a TGuide S96 Magnetic Soil/Stool DNA Kit (Tiangen Biotech (Beijing) Co., Ltd., Beijing, China) according to the manufacturer’s instructions. The hypervariable V3–V4 region of the bacterial 16S rRNA gene was amplified using the primer pairs 338F: 5′-ACTCCTACGGGAGGCAGCA-3′ and 806R: 5′-GGACTACHVGGGTWTCTAAT-3′. PCR products were analyzed on agarose gels and purified using an Omega DNA purification kit (Omega Inc., Norcross, GA, USA). The purified PCR products were collected, and the paired-end sequencing (2 × 250 bp) was performed on an Illumina Novaseq 6000 platform.

The raw reads were quality-filtered using Trimmomatic v0.33 [28], followed by primer removal with Cutadapt v1.9.1 [29]. Paired-end assembly and chimera filtering were performed in USEARCH v10 [30] and UCHIME v8.1 [31], respectively. Operational taxonomic units (OTUs; 97% similarity) were classified against the SILVA 138.1 database [32] using QIIME2’s Naive Bayes classifier (70% confidence, v2020.6). Alpha-diversity (Shannon, Simpson, and Chao1) and β-diversity (unweighted UniFrac PCoA) were calculated. LEfSe (LDA > 4.0) was used to identify differentially abundant taxa, and PICRUSt2 v 2.3.0 was used to predict KEGG pathways [33].

### 2.6. Statistical Analysis

All analyses were based on directly measured raw data, with results expressed as mean ± SD. Normality (Shapiro–Wilk test) and homoscedasticity (Levene’s test) were verified in SPSS 28.0. Intergroup comparisons were performed using one-way ANOVA followed by Tukey’s post hoc test or Kruskal–Wallis H-tests. Intragroup differences were assessed via Student’s *t*-test. Two-way ANOVA was employed to examine the interaction between daily UVB irradiation dose and exposure duration. Microbiome analyses included PERMANOVA (Adonis) [27], Welch’s *t*-test (STAMP), and Spearman correlation (microbiota vs. host parameters). Prior to conducting the Spearman correlation analysis and generating the heatmap, all data were standardized using Z-score normalization. Significance was set at *p* < 0.05 after FDR correction.

## 3. Results

### 3.1. UVB-Induced Histological Alterations in Xenopus

**Figure 1:**
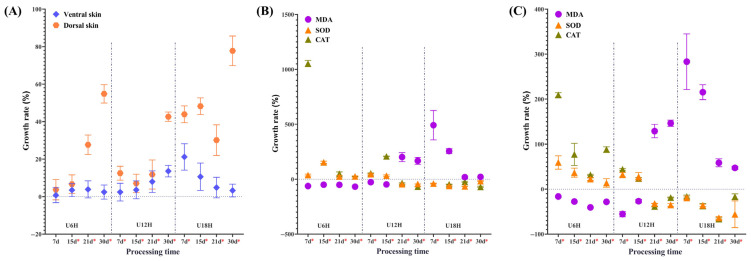
Dose-dependent effects of UVB irradiation on the immune stress responses in *Xenopus* tissues. (**A**) Skin, (**B**) liver, and (**C**) intestinal immune activity expressed as the relative change (%) compared with incandescent lamp controls. Asterisks denote significant differences between the UVB-treated and control groups (* *p* < 0.05 by two-tailed Student’s *t*-test). Error bars represent SEM.

Histological analysis indicated that skin thickness in *Xenopus* increased significantly with prolonged UVB exposure time (*p* < 0.05). Furthermore, higher daily cumulative UVB irradiance led to an earlier onset and more severe degree of skin thickening (Figure 1A). Two-way ANOVA further confirmed a significant interaction between DCI and exposure duration (ventral skin: F = 18.226, dorsal skin: F = 29.483, *p* < 0.01). As the dorsal skin received direct irradiation and thus a substantially higher cumulative UVB dose compared to the shielded ventral side, it exhibited a more pronounced thickening response. Consequently, based on this distinct dose- and location-dependent effect, the dorsal skin was selected for all subsequent analyses as representative of the skin organ.

**Figure 2:**
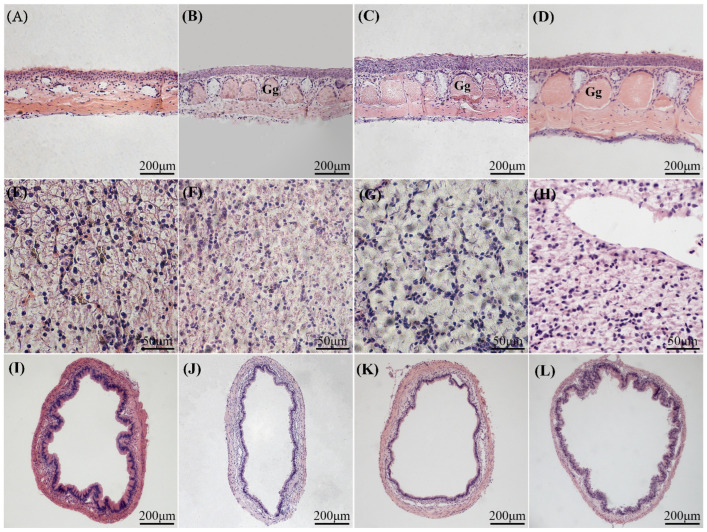
Histopathological alterations in *Xenopus* skin, liver, and intestine following chronic UVB exposure (day 30). (**A**–**D**) Magnified skin sections (10×) showing structural integrity of (**A**) the U6H incandescent lamp control (day 7), (**B**) U6H UVB, (**C**) U12H UVB, and (**D**) U18H UVB groups. Granular glands are indicated by Gg. (E–H) Magnified hepatic sections (40×): (**E**) control (day 7), (**F**) U6H UVB, (**G**) U12H UVB, and (**H**) U18H UVB. (**I**–**L**) Magnified intestinal sections (10×): (**I**) U6H UVB, (**J**) U12H UVB, (**K**) U18H UVB, and (**L**) U18H control. All sections were stained with hematoxylin-eosin (H&E).

The duration of UVB exposure and the daily cumulative irradiance (DCI) significantly affected the histological structure and integrity of both the skin (Figure 2A–D) and the liver (Figure 2E–H) in *Xenopus*. For the skin tissue, within the four irradiation time phases of the low-DCI U6H group, the skin exhibited a notable thickening on the 15th day of irradiation, increasing by 6.60 ± 4.98% compared with the control group (*t*-test: *p* < 0.05; similarly hereafter), and the integrity of the tissue structure was maintained in a satisfactory state. In the medium-DCI U12H group, the epidermis was significantly thickened on the 7th day of irradiation (growth rate: 12.49 ± 3.72%, *p* < 0.05), while the connection between the epidermis and dermis remained intact. In contrast to the U6H group, the granular glands within the skin were augmented after 30 days of irradiation. In the high-DCI U18H group, the epidermis was thickened by 43.93 ± 4.49% on the 7th day of irradiation (*p* < 0.05), and the epidermis and dermis were distinctly separated after 15 days. Furthermore, the granular glands within the skin were markedly larger after 30 days of irradiation than those of the low-DCI U6H and medium-DCI U12H groups. Regarding the liver tissues, the liver tissue morphology in the low-DCI U6H group remained normal at each time point, featuring a clear hepatic lobule structure, regular nuclear shapes, uniform cytoplasm, and no vacuolization. In the medium-DCI U12H group, the hepatic cords were loosely arranged, and the intercellular spaces were expanded on the 30th day of irradiation. In the high-DCI U18H group, the hepatic lobule structure was disordered and extremely swollen, with large fat vacuoles, an increased incidence of cellular steatosis or necrosis, and a decreased nuclear–cytoplasmic ratio as early as the 15th day of irradiation.

**Figure 3:**
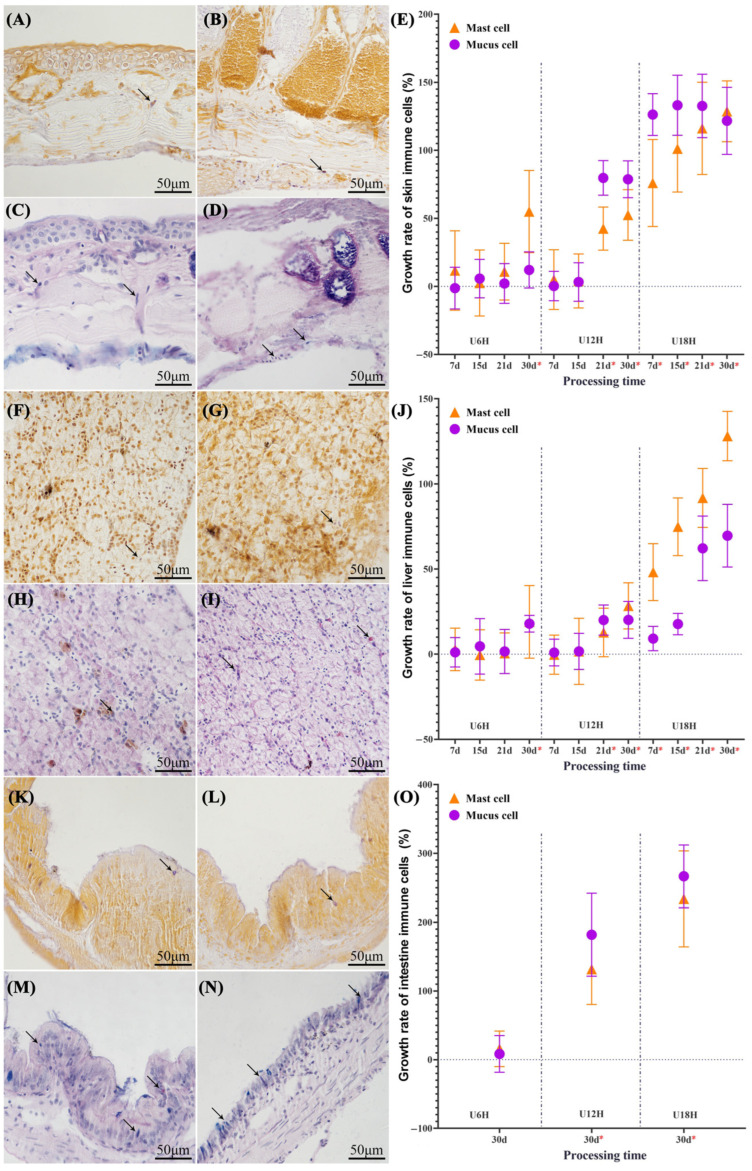
UVB-induced redistribution of immune cells in *Xenopus* tissues (U18H treatment, Day 30). Tissue sections of skin (**A**–**D**), liver (**F**–**I**), and intestine (**K**–**N**) illustrate the distribution of immune cells under different treatments: (**A**,**F**,**K**) mast cells in the incandescent lamp control group; (**B**,**G**,**L**) mast cells in the UVB-treated group; (**C**,**H**,**M**) mucous cells in the incandescent lamp control group; (**D**,**I**,**N**) mucous cells in the UVB-treated group. Mast cells were identified by aldehyde fuchsin-orange G staining, and mucous cells were stained with AB-PAS. (**E**,**J**,**O**) Growth rates of the two types of immune cells in skin, liver, and intestine across different UVB treatment groups with increasing exposure days, respectively. * *p* < 0.05 (two-tailed *t*-test). Scale bars: 50 µm (40× magnification). Arrows indicate stained mast cells/mucous cells.

UVB exposure induced dose- and time-dependent modulation of mast cell and mucous cell distribution in the skin (Figure 3A–E) and liver (Figure 3F–J), demonstrating distinct tissue-specific responses. The results indicated that with increasing DCI and prolonged exposure duration, the densities of both effector cell types showed a synchronized upward trend in the target organs. There was a positive correlation between immune cell density and dorsal skin thickness (Spearman; *p* < 0.05). The high-DCI group (U18H) triggered the earliest and most pronounced cellular proliferation. The densities of mucous cells (↑ 126.31 ± 15.35%) and mast cells (↑ 76.00 ± 31.95%) in the skin tissue showed significant differences starting from the 7th day of intervention (*p* < 0.05). The tests on liver tissues revealed a similar pattern, with the densities of mucous cells (↑ 9.18 ± 7.12%) and mast cells (↑ 48.20 ± 16.72%) increasing synchronously (*p* < 0.05). In contrast, the latency for significant cellular responses was 21 days in the medium-DCI group (U12H) and 30 days in the low-DCI group (U6H) (*p* < 0.05). Two-way ANOVA further revealed a significant interaction between DCI and exposure duration (skin: mucous cells F = 23.954, *p* < 0.01; mast cells F = 2.097, *p* = 0.059; liver: mucous cells F = 14.195, *p* < 0.01; mast cells F = 8.317, *p* < 0.01).

Increasing DCI of UVB exposure significantly affected the histomorphology of the large intestine (Figure 2I–L) and the distribution of immune cells (Figure 3K–O) in *Xenopus*. Comparative analysis on day 30 post-irradiation revealed a gradient of mucosal structural alterations across DCI groups. Compared with the control group, the low-DCI group (U6H) had an intact intestinal morphology, showing no significant deviation in mucosal fold count (*p* = 0.412) or effector cell density compared with controls (↑ mucous cells: 8.43 ± 26.65% vs. mast cells: 15.83 ± 25.89%; *p* > 0.05). The U12H group displayed early pathological changes such as a reduction in the number of mucosal folds and significant increases in the densities of mucous cells (181.82 ± 60.25%) and mast cells (131.67 ± 51.34%) (*p* < 0.05). Meanwhile, the high-DCI U18H group displayed typical structural damage characteristics manifested as a nearly complete loss of mucosal folds, accompanied by the proliferation of both types of effector cells (↑ 266.59 ± 45.62% vs. 233.83 ± 69.70%, *p* < 0.05).

### 3.2. UVB-Modulated Oxidative Stress and Immune Function

The skin and liver exhibited distinct time- and dose-dependent responses in redox homeostasis to varying UVB exposure intensities (Figure 1B,C). Two-way ANOVA further confirmed significant interactions between DCI and exposure duration (skin tissue: MDA F = 52.574, SOD F = 165.597, CAT F = 2183.278, *p* < 0.01; liver tissue: MDA F = 96.219, SOD F = 3.517, CAT F = 54.553, *p* < 0.01). Specifically, the low-DCI group (U6H) showed significantly reduced MDA content (skin: ↓67.98 ± 2.50%; liver: ↓28.32 ± 0.97%) and elevated antioxidant enzyme activities (skin: SOD ↑24.28 ± 5.87%, CAT ↑25.34 ± 1.26%; liver: SOD ↑13.74 ± 9.57%, CAT ↑88.09 ± 5.89%) by day 30, with all changes being statistically significant (*p* < 0.01). In contrast, the high-DCI group (U18H) displayed a significant accumulation of MDA (skin: ↑20.85 ± 2.96%; liver: ↑47.16 ± 3.34%, 30 days, *p* < 0.01) coupled with antioxidant suppression (skin: SOD ↓43.21 ± 1.07%, CAT ↓68.43 ± 1.20%; liver: SOD ↓35.13 ± 2.90%, CAT ↓18.71 ± 0.97%, *p* < 0.01). Notably, the medium-DCI group (U12H) displayed a biphasic response, characterized by an initial activation of the antioxidant system (days 0–15) that transitioned to overt oxidative damage in the later phase (days 16–30).

### 3.3. UVB-Driven Restructuring of the Microbiota

**Figure 4:**
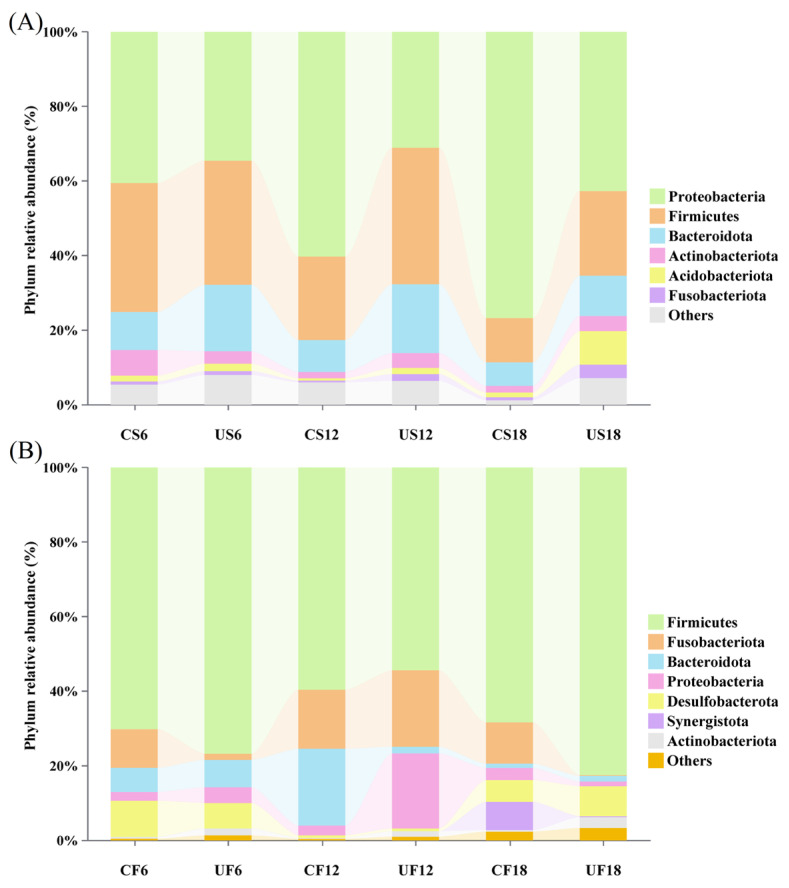
UVB-induced remodeling of *Xenopus* symbiotic microbiota. (**A**) Phylum-level composition of the skin microbiota. (**B**) Intestinal microbiota profiles.

The results of 16S rRNA sequencing of *Xenopus* skin and gut samples yielded 1,412,152 and 1,375,134 quality-filtered reads, respectively (78,452.89 ± 3415.67 vs. 76,396.33 ± 6619.56 per sample), clustered into 1235 (skin) and 1144 (gut) OTUs (555.83 ± 173.11 vs. 401.78 ± 121.11 per sample). For the skin microbiota, a total of 22 phyla, 43 classes, 124 orders, 262 families, and 563 genera were identified. Dominated by Proteobacteria (47.64 ± 20.08%), Firmicutes (26.88 ± 11.79%), and Bacteroidota (12.03 ± 5.97%), accounting for 86.55% (Figure 4A). The α-diversity index results showed that among the three diversity indices (Shannon, Simpson, and Chao1), only the U18H treatment group had a significant difference in Shannon index (*t*-test, *p* < 0.05) (Table 1). For the gut microbiota, a total of 21 phyla, 41 classes, 121 orders, 241 families, and 506 genera were identified. The microbiota was dominated by Firmicutes (68.61 ± 19.34%), Fusobacteriota (9.91 ± 14.41%), and Bacteroidota (6.49 ± 10.43%), accounting for 85.01% (Figure 4B). There were no changes in α-diversity across treatments (CF vs. UF, *p* > 0.05).

**Figure 5:**
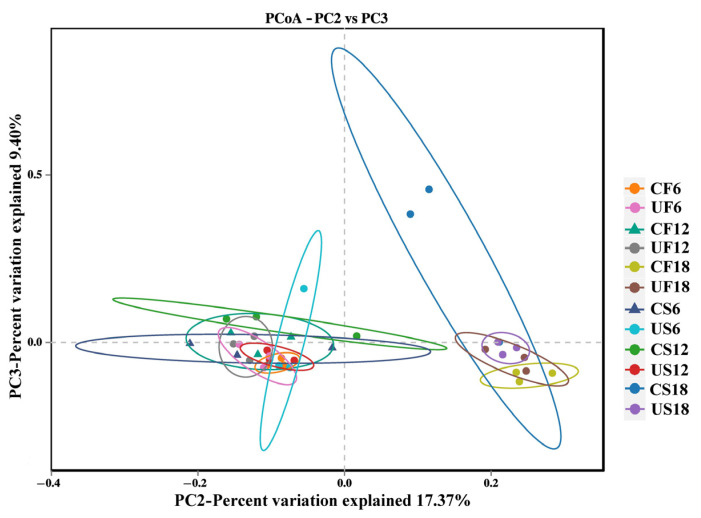
Principal Coordinate Analysis (PCoA) of the symbiotic microbiota in *Xenopus* based on unweighted UniFrac distance (OTU level). Each point represents an individual sample, colored by experimental group. The distance between points reflects the mag-nitude of dissimilarity in microbial community composition.

**Figure 6:**
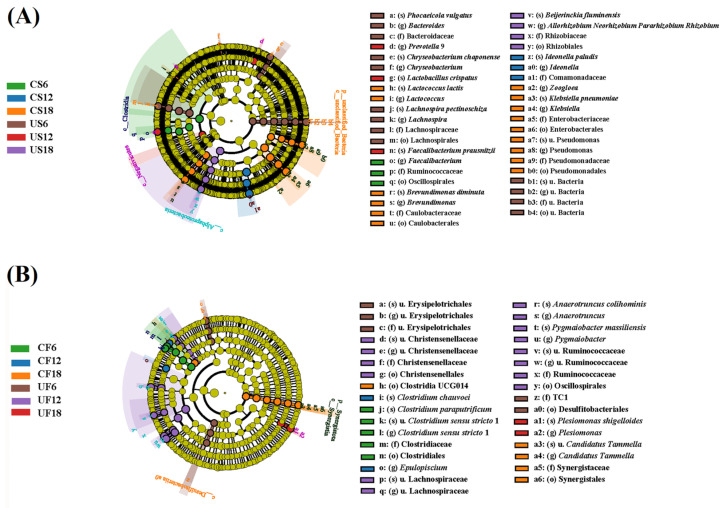
Cladogram of differentially abundant symbiotic microbiota across UVB-DCI treatment groups (genus/species level) by LEfSe analysis. (**A**) Skin microbiota. (**B**) Gut microbiota. Yellow nodes rep-resent taxa with no significant differences among groups. Colored branches and their corresponding nodes indicate group-specific microbial biomarkers. The abbreviation “u.” denotes taxonomically unclassified taxa.

Principal coordinate analysis (PCoA) at the OTU level demonstrated that UVB exposure at varying intensities exerted significant influences on the symbiotic microbial community structure of *Xenopus* (Figure 5). The results demonstrated no significant clustering separation in β-diversity (unweighted UniFrac distance) between the low- and medium-DCI UVB groups, indicating that UV exposure within this range did not breach the homeostatic threshold of symbiotic microbiota. However, the high-DCI group (U18H) exhibited marked inter-group divergence from the lower-DCI cohorts (PERMANOVA, d*f* = 11, R^2^ = 0.63, *p* < 0.05), suggesting structural specificity induced by intense UVB irradiation. Subsequent analysis of the dominant genera (>0.2% relative abundance) identified 89/159 skin-derived and 12/92 gut-derived taxa with significant abundance alterations (*t*-test, *p* < 0.05), exceeding the values of the other treatment groups by orders of magnitude. LEfSe analysis revealed group-specific enrichment of microbial taxa across different daily cumulative UVB irradiance treatments (Figure 6). In the skin microbiota of the U6H group, several genera with recognized anti-inflammatory properties, including *Phocaeicola vulgatus*, *Chryseobacterium*, *Faecalibacterium*, and *Lachnospira*, were significantly enriched (Figure 4 A). The U12H group displayed increased abundances of *Prevotella*_9 (US6 vs. US12: 3.11 ± 1.66% vs. 10.41 ± 6.01%) and *Lactobacillus crispatus* (1.25 ± 0.88% vs. 7.39 ± 8.58%) compared with the U6H group (*p* < 0.05), with concurrent gut-specific enrichment of *Plesiomonas shigelloides* in the UF12 group. High-DCI UVB resulted in cutaneous expansion of the *Allorhizobium–Neorhizobium–Pararhizobium–Rhizobium* complex (US12 vs. US18: 0.21 ± 0.04% vs. 5.20 ± 2.38%, *p* < 0.05) while inducing multi-taxa synergy in the gut microbiota (UF18) through significant enrichment of Christensenellaceae, Lachnospiraceae, *Anaerotruncus colihominis*, *Pygmaiobacter massiliensis*, and Ruminococcaceae (*p* < 0.05), signifying their pivotal roles in community restructuring. Notably, the U18H control group (CS18, 18 h/d incandescent exposure) exhibited marked proliferation of opportunistic pathogens, including *Lactococcus lactis*, *Klebsiella pneumoniae*, *Pseudomonas*, and *Brevundimonas diminuta* (*p* < 0.05).

**Figure 7:**
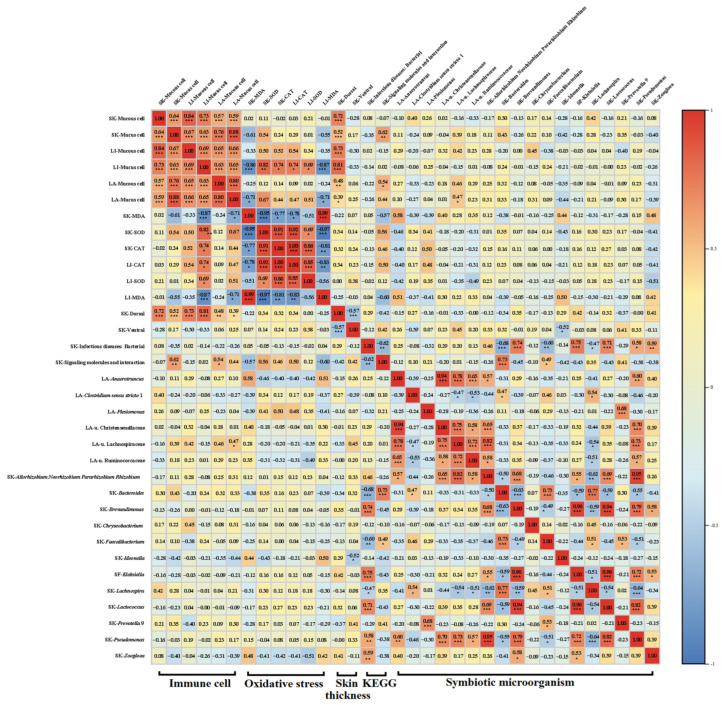
Spearman correlation network of UVB-induced structural and immunological alterations. Integrated analysis of skin histoarchitecture (H&E), immune activity parameters, immune cell distributions, KEGG pathways, and microbiota composition. Correlation significance: *** *p* < 0.001, ** *p* < 0.01, and * *p* < 0.05 (false discovery rate-adjusted). The color gradient represents the strength and direction of the Spearman correlation coefficient, ranging from blue (negative correlation) to red (positive correlation).

Metabolic functional analysis based on the KEGG database revealed that long-term high DCI UVB exposure (U18H) significantly reshaped the functional profile of the skin microbiota. Welch’s *t*-test (*p* < 0.01) identified divergent enrichment in two pathways: “Signaling molecules and interaction” (relative abundance: 3%) and “Infectious diseases: Bacterial” (relative abundance: 50%). The US18 group showed elevated “Signaling molecules and interaction” activity (1.18-fold change vs. control, *p* < 0.05), whereas the CS18 controls exhibited heightened “Infectious diseases: Bacterial” pathway engagement (1.10-fold change, *p* < 0.05), positively correlated with opportunistic pathogen abundance (Spearman; *p* < 0.05, Figure 7). In contrast, no significant KEGG pathway alterations were observed in the gut microbiota under the corresponding DCI UVB exposure.

## 4. Discussion

UVB radiation, as a genotoxic environmental stressor, affects amphibian populations through immunosuppression [34]. Our integrative analysis of histomorphology, oxidative stress parameters, and symbiotic microbiota dynamics systematically evaluated the immunotoxic mechanisms of UVB in Xenopus, establishing a multidimensional framework to characterize biological compensatory–decompensatory responses under gradient UVB exposure.

### 4.1. Multi-Dimensional Response Mechanisms of the Gut–Skin Axis to UVB

As core components of the gut-skin axis, the skin and intestine of amphibians serve not only as physical barriers but also as neuroendocrine-immunological hubs, rendering them highly sensitive to environmental changes [34]. Histopathological analysis further revealed that both the DCI and duration of UVB exposure influenced skin architecture and antioxidant responses.

Low-to-moderate DCI of UVB exposure (U6H–U12H) induced mild oxidative stress, characterized by enhanced antioxidant enzyme activities (SOD, CAT) and controlled levels of the lipid peroxidation product MDA in the liver [19]. At this stage, the organism initiated adaptive repair processes, leading to structural remodeling of the skin, including dermal collagen densification and epidermal hyperplasia [35], suggesting the activation of stem cell-mediated repair mechanisms [36]. This process resembles the protective effects of scallop-derived polypeptide in UVB-injured mice [37]. The physiological significance lies in accelerating cell renewal and increasing skin barrier thickness to counteract oxidative damage and maintain tissue homeostasis [38], thereby alleviating structural disruption in the epidermis and dermis and conferring protective resistance. Concurrently, the significant increase in immune cell numbers indicates that mild oxidative stress and minor tissue damage recruit and activate immune cells. Mucous cells contribute to the physical barrier by secreting a protective mucinous layer on the skin surface, potentially with moisturizing and antibacterial properties that help maintain the local microenvironment. Meanwhile, moderately activated mast cells release a range of cytokines and growth factors (e.g., fibroblast growth factor), which in turn promote skin fibroblast proliferation, collagen synthesis, and epidermal thickening, constituting a key regulatory loop in tissue repair [39,40]. The strong Spearman correlation between oxidative stress markers in the skin and liver and mast cell density supports this mechanism and provides new insights into the potential cross-organ effects of UVB radiation. Notably, *Xenopus* exhibits an evolutionarily conserved photoprotection strategy, involving synergistic defense through the expansion of dermal granular glands (exocrine glands) and compensatory epidermal thickening. This mechanism shares functional convergence with the strategy of *Odorrana andersonii*, which secretes small-molecule antioxidant peptides under high-altitude UV conditions [41]. While *O. andersonii* relies on antioxidant peptides to scavenge free radicals, *Xenopus* may enhance local antioxidant capacity by secreting substances such as CAT from granular glands [42], coupled with a thickened epidermal layer that collectively establishes a dual “physical–biochemical” defense system to reduce UVB penetration and oxidative damage.

Day 15 of U12H treatment marked a transition point between low- and high-radiation effects. Once UVB intensity exceeded a critical threshold (U12H–U18H), skin barrier function shifted from compensation to decompensation. In the high-DCI group (U18H), excessive UVB generated massive ROS, overwhelming the endogenous antioxidant capacity and leading to a collapse of the ROS-scavenging system (SOD/CAT activity↓ and MDA↑) and severe oxidative damage [43]. High ROS levels directly attacked cellular structures and the extracellular matrix, causing collagen degradation, evident separation between the epidermis and dermis, and impaired skin junctional integrity [44,45]. The intestinal barrier was also disrupted, pathologically manifested by the loss of mucosal folds [46], rendering deeper tissues more vulnerable. At this stage, the immune response shifted from reparative to inflammatory. Dysregulated oxidative stress and severe tissue damage served as “danger signals,” triggering explosive proliferation and overactivation of mast cells and mucous cells. In particular, mast cells underwent extensive degranulation, releasing potent pro-inflammatory mediators (e.g., histamine, tumor necrosis factor-α), which further enhanced vascular permeability, recruited inflammatory cells, and instigated a local inflammatory storm that amplified tissue damage and exacerbated oxidative stress, forming a vicious cycle [40,47]. This dynamic transition from protective compensation to pathological injury reveals a “double-edged sword” nonlinear characteristic in the physiological response of *Xenopus* to varying degrees of environmental stress: moderate stress serves as a driver of adaptation, whereas excessive stress becomes a source of damage. Interestingly, this threshold effect appears to be governed not merely by total cumulative radiation but jointly by daily irradiance intensity and exposure duration. For instance, although the cumulative irradiance reached 324 kJ/m^2^ in both the U6H group at day 30 and the U12H group at day 15, the physiological alterations were more moderate under medium-to-low daily irradiance with shorter exposure duration. In contrast, under high daily irradiance (e.g., the U18H group), a cumulative irradiance of only 226.8 kJ/m^2^ (day 7)—equivalent to that of the U6H group at day 30—elicited a more acute and decompensated physiological response. These results indicate that daily irradiance intensity plays a critical role in triggering systemic decompensatory reactions.

### 4.2. Microbial Interactions and Gut–Skin Axis Regulatory Mechanisms

Dysbiosis-induced disruption of mucosal immune tolerance has been identified as a critical pathological mechanism affecting skin homeostasis. Recent studies have confirmed significant associations between gut microbiota imbalance and various dermatological disorders, including atopic dermatitis, systemic lupus erythematosus, and psoriasis [48]. Furthermore, this study found that even moderate-DCI UVB (U12H group) could induce suprathreshold damage in non-irradiated organs (e.g., the liver) through a systemic oxidative stress network, as evidenced by phased fluctuations in oxidative stress status (e.g., a surge in MDA levels by 128.9% ± 14.9% on day 21 in the U12H group). These findings suggest that the multi-organ crosstalk mediated by the “gut-skin axis” may operate through the following mechanisms: (1) habitat cross-transfer of microbiota (e.g., environmental dissemination of gut microbes via fecal-epidermal pathways); (2) amphibian-specific behaviors such as skin-sloughing autophagy, which selectively enriches environmental microbes to reshape the gut microbiota [49]; and (3) direct modulation of cutaneous microenvironments by gut-derived metabolites (e.g., SCFAs) and immune regulators [34]. On the other hand, the liver, as an immunometabolic hub, and the gut form the “gut-liver axis” via the portal venous system. UVB radiation may trigger an oxidative stress cascade in the distal liver through this axis. Amphibians typically adapt to varying environmental pressures through interorgan metabolic division of labor (e.g., compensatory upregulation of hepatic SOD/CAT). Our study suggests that UVB radiation may disrupt this balance, and UVB-induced liver injury (e.g., fatty vacuolization) might be linked to intestinal inflammation or dysbiosis—specifically, the influx of lipopolysaccharide produced by certain Gram-negative bacteria (e.g., Lachnospiraceae), which can stimulate immune cells to overproduce pyrogenic inflammatory cytokines, leading to immune overactivation [50]. Similar mechanisms have been widely reported in cirrhosis models [51], suggesting that UVB may mimic the chronic liver disease microenvironment and induce a similar vicious cycle of “leaky gut–liver injury,” thereby providing new evidence for environmental stress-induced gut-liver axis impairment. Furthermore, we observed a transition from compensation to decompensation in the body’s antioxidant defense system. This dynamic process aligns with findings in *Rana kukunoris* [52] and could serve as a biomarker for assessing cumulative radiation damage, offering a new perspective on the systemic hazards of environmental radiation.

Our findings further elucidate the spatiotemporal regulatory characteristics of UVB radiation on the gut-skin axis. The delayed structural changes in the gut microbiota (PCoA, R^2^ = 0.52) compared to the skin microbiota (R^2^ = 0.50) may stem from UVB interference with skin mast cell activation and the normal migration of dendritic cells to mesenteric lymph nodes [53,54]. This mechanism would explain the significant increase in skin immune cells in the U6H group at day 30 (Figure 3E) versus minimal intestinal changes (Figure 3O), although this hypothesis requires further experimental validation.

In the low/medium DCI exposure groups, although the α- and β-diversity of symbiotic microbiota did not exceed homeostatic thresholds, an increasing trend in overall abundance was observed, suggesting a potential role in enhancing barrier function and maintaining host–microbe symbiotic balance. Specifically, the proliferation of *Faecalibacterium* and *Phocaeicola vulgatus* in the low-DCI group (U6H) may be associated with enhanced SCFA metabolism, thereby strengthening barrier integrity by reducing levels of pro-inflammatory factors such as interleukin-6 and tumor necrosis factor-α [55,56,57]. In the U12H group, the enrichment of *Prevotella*_9 might modulate intestinal barrier function via mucin degradation [58], while *Lactobacillus crispatus* may inhibit pathogen colonization through the secretion of antibacterial peptides, synergistically regulating local and systemic immunity [59]. In this study, aberrant enrichment of *Bacteroides* in *Xenopus* skin (*p* < 0.01) suggested its potential to disrupt the microenvironment via inflammatory mediation and niche competition. However, the gradual decline of *Bacteroides* with increasing UVB intensity offers new insights into the anti-inflammatory mechanisms of phototherapy [60]. These findings align with the gut–skin axis theory, indicating that under low-level UVB radiation, the animal organism can initiate adaptive responses through multiple pathways: not only via synchronized increases in mast cell density in both the skin and intestine, along with oxidative stress activation along the gut-liver axis, but also through the involvement of symbiotic microbiota and their metabolites (e.g., SCFAs) in the immunomodulation of distal organs.

Significant differences in β-diversity of skin and gut microbiota were observed only in the high-DCI group (U18H), indicating that high cumulative UVB exposure may disrupt microbial homeostatic thresholds and drive community restructuring [55]. This pattern is consistent with the effects of altitudinal gradients on symbiotic microbiota in amphibians [61], where high-altitude environments enhance host adaptability by reducing microbial α-/β-diversity, and UVB may mimic such selective pressures to remodel microbial function. Under high-DCI UVB stress, the notable increase in Ruminococcaceae—whose metabolite isoamylamine may impair host cognitive capacity [62]—could contribute to reduced survival. The abnormal proliferation of Christensenellaceae, which produces volatile probiotic metabolites and secondary bile acids, may be linked to host metabolic and anti-inflammatory responses, underscoring the profound impact of UVB-induced gut–skin axis disruption on microbial colonization patterns [63]. Furthermore, the enrichment of opportunistic pathogens (e.g., *Klebsiella pneumoniae* and *Pseudomonas*) in the high-DCI control group (CS18) suggests that photoperiod alteration alone may disrupt microbial balance by interfering with circadian rhythms [64], thereby predisposing amphibians to infection and disease and initiating a “pathogen–inflammation” vicious cycle [65,66]. In natural settings, the extended photoperiod in high-latitude regions may intrinsically influence the structure and function of symbiotic microbiota, and when coupled with intense UVB radiation, could further compromise amphibian survival.

KEGG analysis revealed that UVB exposure significantly up-regulated the “Signaling Molecules and Interaction” pathway (1.18-fold, *p* < 0.05), suggesting that microbiota may enhance stress resistance through quorum sensing mechanisms [67]. This phenomenon resembles the adaptive response of the gut microbiota in marine copepods under seasonal environmental stress [68], where microorganisms optimize resource utilization through functional redundancy and metabolic division of labor. From an evolutionary perspective, UV-protective genes (e.g., photolyase) may be horizontally transferred between symbiotic microbiota and the host in amphibians, facilitating co-adaptation to environmental pressures [69]. Furthermore, microbial restructuring coupled with metabolic specialization (such as in pigment synthesis and quorum sensing symbiosis) enables optimal resource allocation, thereby enhancing the environmental adaptability of the host-microbiota complex system [69].

However, microbial remodeling may represent a double-edged sword. This effect is also observed in *Batrachochytrium dendrobatidis* infection models, where symbiotic microbiota can enhance host survival by antagonizing pathogens; however, environmental stress can disrupt microbial balance and increase infection risk [70]. In terms of immunometabolic regulation, SCFAs can mitigate UV-induced skin inflammation [55], thereby promoting barrier repair and skin thickening, which limits pathogenic bacteria adhesion and invasion [71]. On the other hand, although the UVB-exposed groups exhibited greater stress resistance compared to controls, UV-induced impairment of the skin barrier may facilitate the invasion of opportunistic pathogens such as *Pseudomonas* (relative abundance in CS18 was 791.47 times that in CS12), thereby enriching the “Infectious Diseases: Bacterial” pathway and establishing a defense-pathogenesis dynamic equilibrium [72]. The high enrichment level of this pathway (>50%) reflects a shift in the microbial community from a symbiotic state toward a competitive state. Notably, the greater enrichment of the “Infectious diseases: Bacterial” pathway (fold change = 1.10, *p* < 0.05) in the skin microbiota of the high-DCI control group (CS18) suggests that skin microbiota not treated with UVB may possess higher pathogenic potential. This phenomenon is highly concordant with the enrichment of opportunistic pathogens such as *Klebsiella pneumoniae* and *Pseudomonas*. Pathogenic bacteria and their products (such as lipopolysaccharide) can induce the host to generate excessive ROS, thereby intensifying oxidative damage and disrupting the tissue barrier [73]. These results suggest that UVB irradiation significantly inhibited the activity of this pathway. Ultraviolet rays could weaken the virulence of opportunistic pathogens through direct bactericidal effects or interference with the quorum sensing system. This implies that under long-term UV exposure, the skin microbiota of amphibians may undergo co-evolution to optimize their symbiotic relationship with the host.

In summary, our multi-organ analysis demonstrates a cascade effect of UVB radiation: local skin establishes primary defense through structural remodeling and immune regulation; breaches of the gut barrier trigger metabolic dysregulation; and gut–liver axis dysfunction culminates in systemic injury. High cumulative UVB exposure modulates the structure and function of symbiotic microbiota, and the observed changes in microbial composition and architecture suggest that UVB may exacerbate ecological adaptation risks through host-microbe interactions. These findings advance our understanding of the physiological impacts of UVB on amphibians and provide a methodological framework for related studies.

## 5. Conclusions

This study demonstrates that UVB radiation, through its cumulative effects, induces oxidative damage and immune dysregulation, systematically disrupting the gut-skin axis barrier function in amphibians. Under low-DCI (U6H), the activation of the SOD/CAT-MDA compensatory defense system and the enrichment of anti-inflammatory microbiota delayed pathological progression. In contrast, high-DCI UVB exposure (U18H) triggered decompensation of the redox defense system in symbiotic microbiota and the collapse of β-diversity thresholds, thereby exacerbating the risk of systemic inflammation. To address increasing global UV threats to amphibians, we have proposed multidimensional conservation strategies. (1) Habitat management: Establish vegetation shade belts in high-exposure zones (e.g., alpine wetlands) to reduce the UVB flux. (2) Ecological monitoring: Track population dynamics and microbiome shifts in ozone-depleted/high-altitude habitats to develop early-warning indicators. (3) Captive breeding: administer probiotic supplements to restore gut–skin axis homeostasis, suppress opportunistic pathogens, and implement UVB gradient acclimation to enhance juvenile resilience. Current limitations include the laboratory model’s failure to fully recapitulate cumulative field exposure and ecological interactions, along with unresolved quantitative mechanisms of gut–skin axis metabolite regulation. Future research should integrate remote sensing (UVB flux dynamics) with multi-omics analyses to construct cross-scale ecological models of photostress adaptation, thereby informing adaptive management frameworks for amphibian conservation.

## Figures and Tables

**Table 1:** Comparative Analysis of α-Diversity Indices in Skin and Gut Microbial Communities.

Group	Chao1	Simpson	Shannon
CS6 vs. US6	552.00 ± 122.42 vs. 614.34 ± 163.48	0.92 ± 0.11 vs. 0.98 ± 0.01	6.68 ± 1.72 vs. 7.41 ± 0.66
CS12 vs. US12	488.58 ± 140.23 vs. 696.22 ± 69.76	0.79 ± 0.22 vs. 0.97 ± 0.02	4.96 ± 0.57 vs. 7.09 ± 0.97
CS18 vs. US18	445.71 ± 268.51 vs. 755.52 ± 16.20	0.83 ± 0.10 vs. 0.99 ± 0.01	4.67 ± 1.15 vs. 7.92 ± 0.39 *
CF6 vs. UF6	418.89 ± 23.82 vs. 489.70 ± 59.76	0.85 ± 0.11 vs. 0.89 ± 0.10	4.58 ± 0.78 vs. 5.28 ± 0.88
CF12 vs. UF12	330.74 ± 46.82 vs. 423.69 ± 57.63	0.78 ± 0.03 vs. 0.83 ± 0.07	3.31 ± 0.32 vs. 3.86 ± 0.77
CF18 vs. UF18	695.96 ± 124.94 vs. 500.72 ± 82.17	0.93 ± 0.02 vs. 0.91 ± 0.03	5.04 ± 0.05 vs. 4.80 ± 0.67

* *p* < 0.05 was considered statistically significant.

## Data Availability

All microbial sequences are available from the NCBI under accession number PRJNA1198713.
